# Neurocognitive profiles of learning disabled children with neurofibromatosis type 1

**DOI:** 10.3389/fnhum.2014.00386

**Published:** 2014-06-06

**Authors:** Miladys Orraca-Castillo, Nancy Estévez-Pérez, Vivian Reigosa-Crespo

**Affiliations:** ^1^Provincial Centre for Medical GeneticsPinar del Río, Cuba; ^2^Department of Developmental Cognitive Neuroscience, Cuban Centre for NeuroscienceHavana, Cuba

**Keywords:** neurofibromatosis type 1, specific learning disabilities, developmental dyscalculia, developmental dyslexia, phonological dyslexia, surface dyslexia, arithmetic dysfluency, gender ratio

## Abstract

Neurofibromatosis 1 (NF1) is a genetic condition generally associated with intellectual deficiency and learning disabilities. Although there have been groundbreaking advances in the understanding of the molecular, cellular, and neural systems underlying learning deficits associated to NF1 in animal models, much remains to be learned about the spectrum of neurocognitive phenotype associated with the NF1 clinical syndrome. In the present study, 32 children with NF1 ranging from 7 to 14 years were evaluated with neurocognitive tests dedicated to assess basic capacities which are involved in reading and mathematical achievement. Deficits in lexical and phonological strategies and poor number facts retrieval were found underlying reading and arithmetic disorders, respectively. Additionally, efficiencies in lexical/phonological strategies and mental arithmetic were significant predictors of individual differences in reading attainment and math. However, deficits in core numeric capacities were not found in the sample, suggesting that it is not responsible for calculation dysfluency. The estimated prevalence of Developmental Dyscalculia was 18.8%, and the male:female ratio was 5:1. On the other hand, the prevalence of Developmental Dyslexia was almost 3 times as high (50%), and no gender differences were found (male: female ratio = 1:1). This study offers new evidence to the neurocognitive phenotype of NF1 contributing to an in depth understanding of this condition, but also to possible treatments for the cognitive deficits associated with NF1.

## Introduction

Neurofibromatosis 1 (NF1) is an autosomal dominant disease caused by mutations of the NF1 gene, a tumor suppressor gene, on chromosome 17. Almost all affected individuals exhibit multiple cafe'au lait spots and cutaneous neurofibromas. Less frequent but more serious manifestations include central nervous system gliomas, plexiform, neurofibromas, and malignant peripheral nerve sheath tumors (Rasmussen and Friedman, 2013).

Learning, cognitive, and neurobehavioral deficits are highly prevalent in this condition (Acosta et al., 2012). As a single gene disorder, NF1 provides a unique genetic model to identify and analyze the molecular and cellular bases underlying cognitive dysfunction. Thus, this disorder has received the full attention of the scientific community dedicated to the translation of basic or bench science, to bedside clinical practice or dissemination to population-based community interventions (translational science) (Acosta et al., 2012).

Nevertheless, the marked clinical variability described in the syndrome suggests that NF1 is composed of numerous distinct diseases, each defined by factors including patient age, patient sex, the timing of NF1 inactivation, the specific cell type, genomic modifiers, and microenvironmental influences. No single molecular abnormality seems to underlie all of the cognitive dysfunction observed in children with NF1. Rather, the specific collection of cognitive and behavioral deficits exhibited by any given child with NF1 probably reflects the relative contributions of multiple cellular and molecular defects (Diggs-Andrews and Gutmann, 2013). Considering this, it has been suggested to deal with the cognitive deficits in children with NF1 by integrating therapies specifically designed to target the biochemical and neurochemical abnormalities unique to each child (Diggs-Andrews and Gutmann, 2013).

Children with NF1 usually exhibit low intellectual capacities. Attention Deficit and/or Hyperactivity Disorders (ADHD), depression, poor school attainment and postschool outcomes are frequently reported as well (Mazzocco, 2001; Billingsley et al., 2004; Payne et al., 2010; Rasmussen and Friedman, 2013). The cognitive impairments more frequently described include specific learning disabilities (SLD)—such as Developmental Dyslexia (DL) and Developmental Dyscalculia (DD)—visuo-spatial orientation, and attention deficits, language acquisition, spatial memory and executive functions defects (Billingsley et al., 2004; Payne et al., 2010).

There are various genetic syndromes which exhibit SLD. Together with NF1, Turner, Fragile X, and Velocardiofacial syndromes are the most studied (Mazzocco, 2001; Molko et al., 2004; Murphy and Mazzocco, 2008). SLD have been reported in 30–70% of children with NF1 (Hyman et al., 2005; Acosta, 2007). Nevertheless, the diagnosis of SLD has mainly focused on IQ-achievement discrepancy (Hyman et al., 2005; Watt et al., 2008). The discrepancy model has been criticized for numerous authors. Over the past 30 years dozens of research articles have provided empirical evidence of the problems inherent to the IQ-achievement discrepancy model (Restori et al., 2009). It is now clear that establishing the discrepancy between intelligence and achievement is not particularly useful either for assessment or intervention purposes.

As an alternative point of view, current theories of typical cognitive development postulate that achievement is based on a restricted set of core systems, defined as domain-specific representational primitives that lead and constrain the cultural learning (Spelke and Kinzler, 2007). Thus, the focus of attention has shifted from higher level, school-like arithmetic and reading skills to an analysis of basic capacities, or domain-specific cognitive skills.

According to this approach, DD is considered a congenital and persistent disability in achieving normal levels of arithmetical skills that arise when the specialized number system (Feigenson et al., 2004; Butterworth, 2010) fails to develop normally with corresponding deleterious effects in the acquisition of higher level math skills. Neuroimaging studies of neural foundations of DD compared to controls describe low number-related activations in the Intraparietal Sulcus (an area dedicated to number processing) and the recruitment of more distributed brain regions (possibly reflecting compensatory strategies) (Kaufmann et al., 2011).

Although this approach focuses on core numeric capacities, it also accepts the contribution of other domain-general cognitive processes such as executive functions to arithmetical attainment (Butterworth, 2010). A deficient recruitment of frontal brain regions and supramarginal and postcentral gyrus, areas found to support domain-general processing in typically developing children (Kaufmann et al., 2011) supports the role of domain-general cognitive processes in the numerical cognition.

On the other hand, DL is considered a congenital and persistent disability in reading and comprehension, and exhibits deficits in phonological processing - the awareness of the sound structure of words—and, in some cases, a more fundamental deficit in rapid auditory processing (Temple, 2002). Neuroimaging studies of neural foundations of DL describe underactivation in the left hemisphere inferior frontal gyrus and temporoparietal cortex (underlying deficient phonological processing) and the visual word form area in the left hemisphere occipitotemporal region (important in word recognition) (Richlan, 2012). Inferior frontal white matter decreases in the left frontal and parietal portions of the arcuate fasciculus and other left perisilvian areas have also been associated to the disorder (Silani et al., 2005).

To the best of our knowledge, the identification of DD focused on defective basic numerical capacities and math fluency has not been previously addressed in children with NF1 and only one study evaluated DL subtypes considering phonological and lexical deficits in this genetic disorder (Watt et al., 2008). The present study aims to evaluate a group of children with NF1 in order to determine the presence of DD and DL based on profiles of defective core capacities, using neurocognitive tests specifically designed and standardized for the diagnosis of these SLD. Gender ratios of the detected disorders are also examined in the sample studied. The confirmation of the presence of the disorders in the neurocognitive phenotype of NF1 syndrome would contribute to the development of specific clinical and behavioral interventions for NF1 syndrome. In addition it would contribute to the modeling of the core cognitive and neurobiological foundations of typical and atypical learning of reading and mathematics.

## Materials and methods

### Ethics statement

The study was approved by the Ethics Committee of the Provincial Center for Medical Genetics (PCMG), Pinar del Río, and by the Institutional Review Board of the Cuban Center for Neuroscience and it's in line with the Cuban Science, Technology and Environment Ministry's approved projects. No identity revealing photographs were taken. Written consent was obtained from all parents, and all participants provided verbal assent for all assessments.

### Participants

Thirty two (32) children (14 girls) with NF1 registered and screened by the PCMG in the Pinar del Río province, ranging from 7 to 14 years of age were included [7–8 years old: 5 children (3 girls); 9–10 years old: 7 children (4 girls); 11–12 years old: 10 children (2 girls); 13–14 years old: 10 children (5 girls)]. NF1 was diagnosed according to the criteria described by the National Institutes of Health Consensus Development Conference (Ferner et al., 2007). Among the main clinical signs, children included in the sample exhibited (more than six) pigmented birthmarks typically distributed around the torso and the arms/legs, freckling of the armpits, skeletal abnormalities and (two or more) Lisch nodules and/or neurofibromas.

### Tasks

#### Attainment tests

Two attainment tests were used in order to detect numerical and reading impairments in the classroom. The MAT (Mathematics Attainment Test) and RCAT (Reading and Comprehension Attainment test) are non-standardized curriculum-based measurements developed by researchers at the Ministry of Education (Bernabeu and León, 2003, Pers. Commun.) and employed throughout Cuban schools. The authors created the measures by selecting problem types and texts representing a proportional sampling of the computation and reading skills within the national curriculum.

MAT comprised eight computational problems by each respective grade (2 to 9th). Total score was up to 8 (one per each problem performed correctly). Children were considered to fail the MAT test if obtaining a total score ≤5.

RCAT comprised a text and five comprehension questions related to it. Total score was up to 5 (one per each correct answer). Children were considered to fail RCAT if obtaining a total score ≤3.

#### Core cognitive assessment

Item-timed computerized tasks from two standardized neurocognitive batteries designed in order to evaluate core cognitive processes recruited by numerical processing and reading were used: mental arithmetic, dot enumeration, number magnitude comparison and simple reaction time tasks from the Basic Numerical Battery (BNB) (Reigosa-Crespo et al., 2012) and the word and pseudoword reading task from Batería de Trastornos de la Lectura (BTL) (Reigosa-Crespo et al., 1994).

***Standardization Data of BNB***. BNB was standardized using a stratified random sampling strategy (Pedhazur and Pedhazur-Schmelkin, 1991) which reduces sampling variability by creating relatively homogeneous strata with respect to the dependent variable of interest. this strategy allows the test developer to produce norms with less sampling error as would a simple randomized sample (Crocker and Algina, 1986).

The school-age population (*n* = 11,652) of a municipality of Havana was first split up by grade (2 to 9th) and then, divided into nine strata on the basis of the score obtained on MAT test (0–8). Each stratum was initially treated independently. Thus, children within each stratum were randomly selected and individual estimates (proportions) were obtained. These estimates were then weighted to arrive at an estimate for the population parameters.

The normative sample was comprised by 376 children (188 boys and 188 girls) homogeneously distributed by grade. No significant differences were found between the proportions of individuals randomly selected and the proportions of individuals of general population within each stratum.

Medians of reaction time for correct responses in the numerical tasks were adjusted subtracting each by the median of the simple RT for that participant (adjRTs). Then, an *efficiency measure* (EM) for each task was calculated using adjRTs divided by the proportion of hits (EM = adjRT/Hits). This is an inverse measure, so higher scores represent worse performance. Since EMs did not fit a normal distribution (the data were skewed to the right), a logarithmic transformation of EMs (logEMs) was performed (Reigosa-Crespo et al., 2012).

The norms for the BNB tasks were calculated using the General Regression Model module of Statistica 6.1 software. A regression of logEMs as function of age with a linear and a quadratic component was estimated for each task. The residuals of the regressions were obtained for each individual. The normative parameters for all the tasks were obtained and the individual residual for each test obtained from a given child are used to calculate a Z-score by test, thus allowing the comparison of new subjects with the corresponding (age-appropriate) normative data.

#### Tests description

***Basic Numerical Battery (BNB)***. BNB (Reigosa-Crespo et al., 2012) is a battery of item-timed computerized tests. BNB includes two numerical capacity tests: dot enumeration and number magnitude comparison, and also, a test of mental arithmetic fluency. Each test included practice trials for ensuring the understanding of the instructions. The children always had to give a response by pressing the corresponding key (thus misses were not measurable). Only the keys of the numeric pad (right side of the keyboard) were available for response (excepting the Simple Reaction Time task).

*Simple reaction time*. Children were asked to press the space key as soon as they saw a square in the center of display. The inter-stimulus presentation time was variable (500–1500 ms). Twenty trials were presented. Five practice trials were given before starting the test. Reaction times (RT) were recorded. The simple reaction time test was not analyzed by itself. It was considered a baseline measure of processing speed. The reaction times on all numerical tasks described below were adjusted taking this measure into account.

*Mental arithmetic*. Fifteen simple additions, 15 subtractions and 15 multiplications were presented in three separate blocks. All involved single-digit numbers from 2 to 9. Items were presented on the computer screen (in the form “2+4”). No ties (e.g., 3 + 3, 5 × 5) were presented, and items were not repeated. Two practice trials were given before the start of each block. Children were asked to type in the answer as quickly as they could without making any mistakes. Reaction time was measured with the first key stroke. Hits were also recorded.

*Dot enumeration*. Randomly arranged dots ranging from 1 to 9 were presented on the computer display. Children were asked to estimate the number of dots and to respond as quickly as they could without making mistakes. Eighteen trials were presented altogether, with each number from 1 to 9 being presented twice in a pseudo-random order with the proviso that no item occurred twice in succession. Five practices were given before starting the test. Reaction times and hits were recorded.

*Number Magnitude Comparison*. Children were presented with 25 pairs of digits (numerosities: 1–9, numerical distances: 1–3) on the computer screen. The children were asked to compare the numbers on left-right direction (e.g., 5 < 7, 7 > 5); accordingly, the number in the left side appeared before the number in the right side. Both numbers remained on the screen until the answer was recorded. Five practices were given before starting the test. Reaction Times were recorded by pressing the key corresponding to the answer (1 for “smaller than,” 2 for “bigger than” and 3 for “equal as”). Hits were also recorded.

EM for the three numerical tasks were produced as explained when describing BNB sstandardization procedure. For the mental arithmetic test, the EM scores for each operation (addition, subtraction, and multiplication) were calculated for each child. The mean of these medians for each child was then used as a measure of efficiency on the mental arithmetic test overall. As in a previous study [11], these two measures (RT and proportion of hits) were used because it had been noted that children with low numeracy tend to adopt strategies that produce generally accurate answers but extremely long RT latencies; or they would simply guess quickly, leading to inaccurate answers but short RT latencies. Note that higher EM scores represent worse performance.

Individual Z-score for each test was calculated using the mean and standard deviation (SD) of the residuals of the regressions of EMs as a function of age, estimated from the normative sample.

***Word and pseudoword reading***. This task evaluates the phonological and lexical strategies involved in written word decoding and is included in the standardized BTL battery (Reigosa-Crespo et al., 1994). The authors reported the word and pseudoword reading task exhibited appropriate validity and reliability values (Reigosa et al., 2002).

The children were required to read 30 words balanced by frequency, number of letters and syllables and 30 pseudowords. Each stimulus was presented sequentially. The trial ended after the child responds or after 5000 ms with no response. Ten practice trials were presented initially to ensure that the children understood the task. Responses were verbal and triggered a voice activated key which measured vocal response latencies from the onset of presentation. Errors were recorded by the experimenter.

Median vocal response times (vRT) for correct responses for word and pseudo-words were calculated separately. Then the correponding efficiency measure (EM) was calculated by diving the vRT by the proportion of hits (EM = vRT/Hits). As in the BNB tasks, individual Z-scores for words and pseudo-words were calculated using the mean and standard deviation (SD) of the residuals of the regressions of EMs as a function of age, estimated from the normative sample.

#### Intellectual capacity

Raven's Colored Progressive Matrices (RCPM) test (Raven et al., 1992).

This test was administered as a measure of non-verbal reasoning ability. In this test, a colored pattern is shown with a missing piece. Below the pattern, six pieces, all fitting in the blank but with different patterns are shown. The child has to select the piece that fits in the pattern above. The total number of correct selections was recorded. Each child completed the entire test, consisting of 36 items.

#### Procedure

All children were evaluated at the PCMG. The individual results were compared with standardized values of each test. According to the diagnostic criteria for specific learning disorders proposed in the Diagnostic and Statistical Manual of Mental Disorders (American Psychological Association, 1994), children were classified as follows:

***Developmental dyslexia***. It was determined by failure in both, RCAT test and word and/or pseudoword reading test (a Z-score < 2 SDs), and normal intellectual capacity (score greater that the 5th percentile in the RCPM test).

Concerning DL neurocognitive profile, children were labeled as phonological dyslexics when only their phonological reading skills (assessed with pseudoword reading) were impaired, and surface dyslexics when only their orthographic reading skills (assessed with high-frequency word reading) were impaired; when both were deficient, they were said to have a mixed profile. The cutoff for defining a reading skill as impaired was 2 SD below the mean of the normative group.

***Developmental dyscalculia***. It was determined by failure in MAT test and at least, in one of the BNC tests (a Z-score < 2 SDs in the dot enumeration, magnitude comparison and/or mental calculation tasks) and normal intellectual capacity.

Concerning DD neurocognitive profile, children were labeled as arithmetically dysfluent when their mental arithmetic fluency was deficient (assessed with mental arithmetic task), and core numerically disabled when only their estimation and/or number comparison capacities where deficient (assessed with the corresponding numerical core cognitive tasks).

***Learning disabilities associated with intellectual disability***. It was determined by failure in RCAT test, Z-score < 2 SDs in the word and pseudo-word reading task and/or failure in the MAT test, and/or Z-score < 2 SDs in at least one of the three capacity tests (dot enumeration, numerical magnitude comparison and mental arithmetic), associated with low intellectual capacity (≤5th percentile in RCPM test).

***No Learning Disabilities***. Children with normal academic achievement and intellectual capacities and Z-scores between ± 2 *SD*.

We also tested whether efficiency measures in numerical processing (efficiency for enumeration and number comparison), arithmetic fluency (efficiency for mental arithmetic) and reading (efficiency for reading words and pseudowords) predicted individual variations in math and reading attainment. Generalized linear models were performed for each predictor. In the models, EMs were defined as continuous predictor variables. MAT scores (0–8) and RCAT scores (0–5) were defined separately as dependent variables. The models assumed an ordinal multinomial distribution of dependent variables because they may be ordered as categories.

Male:female (M:F) ratios corresponding to each classification were calculated.

## Results

Learning disabilities were diagnosed in 17 (53.1%) of the 32 children evaluated (Table 1). Co-morbidities between DD and DL were observed in the sample; nevertheless double dissociations between DD and DL were also detected. Ideally, a double dissociation is evinced when at least one case exhibits intact process A and impaired process B, and at least another case exhibits impaired process A and intact process B (Caramazza, 1986; Shallice, 1988). These complementary patterns of deficits, in addition, suggest independent neural networks subserving the processes. Eleven children exhibited DL without any deficiency in number processing and calculation, and one child showed the opposite pattern: DD with spared reading skills.

**Table 1:** **Prevalence of learning disabilities in the sample of children with NF1**.

**Classification**	***N***	Prevalence^*^ (%)	**M:F ratio**
Specific learning disabilities	17	53.1	1.18:1
DL	16	50.0	1:1
DD	6	18.8	5:1
Learning disabilities associated to intellectual disability	6	18.8	1:1
No learning disabilities	9	28.1	2:1

DL, Developmental Dyslexia; DD, Developmental Dyscalculia; N, number of children; M:F ratio, male/female ratio.

* Clinical prevalence calculated based on sample size (32 children).

Among the children who showed only one SLD, different neurocognitive profiles were also observed (Tables 2, 3). Seven of the 11 children diagnosed with DL, presented the mixed subtype and four, the surface (dyslexical) subtype. Interestingly, children with phonological deficits and spared lexical skills were not found. The NF1 children with DD, exhibited selective deficit in mental calculation (arithmetic dysfluency) associated with spared enumeration and magnitude comparison skills.

**Table 2:** **Distribution of learning disabled children with NF1 according to the neurocognitive profile**.

**Classification**	***N***	**(%)**
DL (Mixed subtype)	7	21.9
DL (Surface subtype)	4	12.5
DD (arithmetic dysfluency)	1	3.1
DL (Mixed subtype)/DD (arithmetic dysfluency)	3	9.4
DL (Surface subtype)/DD (arithmetic dysfluency)	2	6.2
Total	17	53.1

**Table 3:** **Cognitive measures in the sample of children with NF1 according to the neurocognitive profile**.

**Measures**	**No LD (***N*** = **9**)**		**DL (***N*** = **11**)**		**DL DD (***N*** = **5**)**		**LD & ID (***N*** = **6**)**	
	**Mean**	***SD***	**Mean**	***SD***	**Mean**	***SD***	**Mean**	***SD***
RT	496.111	204.172	481.818	157.6	453.400	208.211	392.667	122.463
Age	9.667	1.732	11.636	2.111	11.4	1.517	12.5	1.975
RCPM test^+^	22.556	6.346	23.75	3.536	25.4	4.722	13.333	2.338
RCAT^+^	4.111	0.928	2.909	1.3	2.4	1.14	2.167	1.472
MAT^+^	6.625	1.768	3.7	1.829	4.0	1.826	3.0	2.449
Enumeration^*^	2537.906	705.081	2365.341	979.122	3041.657	389.225	2612.081	344.765
Number Comparison^*^	1795.130	390.043	1682.463	435.104	2372.540	1212.735	1988.895	637.409
Mental Arithmetic^*^	3621.818	1511.766	5077.210	4650.242	8544.266	1346.224	5539.682	4096.582
Word reading^*^	2018.754	276.394	2530.147	578.286	2939.580	423.303	3030.208	1298.428
Pseudoword reading^*^	2858.331	397.089	4755.116	1774.514	4858.311	1839.970	4335.839	1278.204

+ Raw scores.

* Efficiency Measures.

DL: Developmental Dyslexia.

DL DD: Developmental Dyslexia and Developmental Dyscalculia.

LD & ID: Learning Disabilities associated to intellectual disability.

No LD: No Learning Disabilities.

SD: Standard Deviation.

Additionally, EM of mental arithmetic were significant predictors of MAT scores [*W*_(1, 8)_ = 4.04, *p* < 0.05]. EMs of reading words and pseudowords were also significant predictors of RCAT scores [*W*_(1, 4)_ = 9.31, *p* < 0.01 and *W*_(1, 4)_ = 3.98, *p* < 0.05 respectively].

Gender ratios suggest that boys and girls had similar probabilities of having DL. In contrast, boys were 5 times more likely to suffer DD than girls.

Another finding of the study is the detection of generalized learning disabilities associated with low intellectual capacity in 18.8% of the NF1 sample. Finally, nine of the NF1 children (28.1% of the sample) did not show learning disorders.

## Discussion

The prevalence of SLD in the sample of children NF1 is in accordance with that reported in international publications, which ranges between 30 and 65% of the affected children (North et al., 1997; Hyman et al., 2005; Ferner et al., 2007). Another study, including cognitive assessments of basic capacities also reported higher proportion of reading, spelling, and mathematics deficits (51%) in children with NF1 in contrast with specific learning disabilities defined by IQ–achievement discrepancies (only 20% of the children). Co-morbidities between Developmental Dyslexia and Dyscalculia were also observed in the sample, in accordance with previous reports (Mazzocco, 2001).

In general, the studies dedicated to the evaluation of core numeric processes and reading skills in genetic syndrome populations are scarce (Mazzocco, 2001; Bruandet et al., 2004; Molko et al., 2004). Most of the current research is focused on the use of academic achievement tests and the observation of a variety of general and specific cognitive deficits, usually disregarding the modular systems involved in cognitive processing; which are increasingly becoming relevant in neurocognitive models of typical and atypical learning (Spelke and Kinzler, 2007).

The unvailability of detailed reports of learning disabilities diagnosed in considering the presence of specific deficits in numeric and arithmetic skills in NF1 does not allow the direct comparison of the results obtained in this study with those of the literature (Mazzocco, 2001; Butterworth and Reigosa-Crespo, 2007; Clements-Stephensa et al., 2008). Nevertheless, the prevalence estimates based on math achievement measures in the general population range from 3 to 14% (median of approximately 6%), (Kosc, 1974; Badian, 1983; Lewis et al., 1994; Gross-Tsur et al., 1996; Desoete et al., 2004). The prevalence of DD found in the NF1 sample is much higher than that, but, together with the presence of the genetic disorder, this could be related to the use of highly specific and sensitive neurocognitive diagnostic techniques.

A previous study (Reigosa-Crespo et al., 2012) using the same neurocognitive tests employed here, reported 9.35% of the general population (children from 2 to 9th grades) exhibited Arithmetic Dysfluency (AD). The results presented here are also higher, but this could be related to the fact there are additional (general domain) cognitive deficits associated to NF1 syndrome that can potencially affect arithmetical processes. Note DD children with NF1 exhibited spared core numeric capacities (arithmetic dysfluency was the only numeric processing impairment detected) which supports the general domain deficits hypothesis.

A variety of numerical and non-numerical cognitive disabilities described in the general population have been described to have an impact in arithmetic performance: inadequate counting-based and retrieval-based strategies from long-term memory (Geary, 1993; Jordan and Montani, 1997; Jordan et al., 2003) as well as low IQ score (Geary et al., 2000). Other domain general processes, generally described as deficient in NF1, such as executive functions (Bull and Scerif, 2001) and visuo-spatial working memory (Wilson and Swanson, 2001) have been also reported to correlate with mathematical abilities.

Regarding the neural foundations of the relevant cognitive processes, the IPS, a key structure underlying number processing is also believed to be involved in visuo-spatial working memory, and visuo-spatial attention (Rotzer et al., 2009). Accordingly, Rotzer et al. (2009) reported DD children exhibit lower behavioral performances in non-numerical visuo-spatial working memory abilities, along with lower activity in the IPS. The authors suggest these poor spatial working memory processes may inhibit the formation of spatial number representations and the storage and retrieval of arithmetical facts. The recruitment of extra-parietal regions (found upon contrasting children vs. adults and children with and without DD) also supports the contribution of general abilities such as working memory, in numeric and arithmetic achievement (Kaufmann et al., 2011).

In addition, a recent translational study revealed working memory deficits in Nf1 mice and in NF1 patients and hypoactivation of related cortical striatal networks (Shilyansky et al., 2010). The studies with NF1 patients showed that the degree of hypoactivation of corticostriatal networks in NF1 was predictive of the degree of impairment in working memory tasks. The convergent cross-species findings suggest hypoactivation of corticostriatal structures, observed in NF1 patients, may be caused by increased GABA(A) receptor signaling, an effect previously reported in fMRI studies of healthy subjects given GABA(A) agonists (Menzies et al., 2007). Additionally, Shilyansky et al. (2010) examined the parallel effects of Nf1 mutations across species in homologous neuronal circuits, using behavioral tasks that specifically address working memory function. They provided evidence that neurofibromin/Ras signaling in both mice and humans regulates working memory by modulating inhibition in prefrontal cortical and striatal networks. NF1 individuals showed significantly reduced neural activity relative to controls in the dorsolateral prefrontal cortex (DLPFC), frontal eye-fields and parietal cortex. Tasks performance was predicted by the activation in the right DLPFC.

Altered inhibition relative to excitation has been reported in other animal models of neurodevelopmental disorders in which corticostriatal hypoactivation and working memory deficits are also found. Working memory deficits have been described in Fragile X (Kwon et al., 2001), and Turner syndrome (Haberecht et al., 2001); disorders in which DD has been also detected as part of the neurocognive phenotype. The working memory deficits in these syndromes were associated with corticostriatal hypoactivation as well.

The present study didn't include domain general cognitive function evaluations, other that intellectual capacity, in order to comply with SLD diagnosis requirements. Future studies in children with NF1 in which SLD are detected should consider these processes, and include working memory function assessment in order to detail the nature of the cognitive profiles described. The design of therapies specific to the biochemical and neurochemical abnormalities unique to each child would benefit significantly from this practice.

Concerning the DL findings, to the best of our knowledge, only one study examined lexical and phonological reading skills in children with NF1 (Watt et al., 2008). The authors reported 67% of the sample exhibited deficits in one or more reading skills. Seventy-five percent of this subgroup exhibited phonological dyslexia. Their findings indicate that a large proportion of children with NF1 may be characterized by a specific difficulty when employing spelling-to-sound rules to assemble a pronunciation sequence.

The present study didn't detect pure phonological deficits in the sample analyzed. This could be related to the fact that Spanish is a transparent language (Seymour et al., 2003). On the other hand, the prevalence of this subtype in the general population in Cuba is also significantly smaller than the corresponding to the dyslexical subtype (Reigosa et al., 2008). Considering the sample size of this study, it is statistically improbable to detect cases exhibiting this subtype.

The double dissociation found here in NF1 children between DD and DL supports the existence of different modular cognitive and neural systems, specialized in reading and numerical processing. From the neuropsychological perspective, evidence of double dissociations between processing deficits would strongly support the relative autonomy of the implicated processes and therefore, of the neural networks underlying them. This is especially relevant considering that NF1 is a genetic condition, which suggests that the development of the circuits subserving these domain-specific processes is highly controlled by genetic mechanisms. These findings add to current neurocognitive modeling of learning and cognitive function in typical neurodevelopment.

Additionally, the analysis revealed that efficiencies in mental arithmetic and lexical/phonological strategies were significant predictors of individual differences in math and reading attainment, respectively. These findings validate the diagnosis criteria used in this study, and also support the current theories of cognitive development that postulate the role of a set of basic capacities on higher level, school-like skills.

The gender ratios related to the presence of SLD are in accordance with reports in NF1 suggesting boys are at significant risk for SLD (Hyman et al., 2006). Some neurobiological evidence could explain this finding. There have been described significantly less leftward asymmetry in the left planum temporale in boys with NF1, associated with poorer reading and math achievement. Significantly smaller surface area and gray matter volume were also found in this area in boys, compared with girls with NF-1 and controls (Billingsley et al., 2002).

The gender ratios related to the presence of DD diagnosed based on arithmetic dysfluency are much higher (5:1) than the corresponding ratios in the general population reported in the prevalence study conducted by Reigosa-Crespo et al. (2012), where no difference was found between boys and girls. On the other hand, the ratios corresponding to DD diagnosed on the basis of core numeric deficits were similarly high (4:1) to the NF1 sample. This contrast in gender preponderance suggests arithmetical dysfluence in NF1 doesn't depend on environmental factors, such as lack of exposure of arithmetical facts or poor schooling (Geary et al., 2000), as could be the case in the (typical) general school-age population. Rather, the cognitive deficits underlying arithmetic dysfluency in NF1 seem to be biologically determined, as in the case of DD associated to pure core numeric deficits.

The M:F ratio found for DL suggests boys with NF1 are as likely as girls to exhibit DL. In contrast, the gender ratio in samples of children with reading difficulties range from 2:1 to 15:1 males to females. It has been suggested that a greater variance of reading performance measures in males may account at least in part for their higher prevalence of reading difficulties as well as for the higher gender ratios reported. However, in research-identified samples, gender ratios are closer to 1:1 (Hawke et al., 2009).

The prevalence of generalized learning disabilities associated with low intellectual capacity in the sample is much higher than that reported in reviewed publications. Between 3 and 8% of people with NF1 show intellectual disability (Ferner et al., 2007; Pride et al., 2010; Acosta et al., 2012). Nevertheless, as in the case of arithmetic dysfluency deficits found in this sample, this could be associated with other cognitive domain general processes affecting intellectual capacity. For instance, there has been reported working memory explains 54% of the variance in the Raven's Colored Progressive Matrices Test (De Ribaupierre and Lecerf, 2006). Additionally, in accordance with previous studies, despite neurocognitive impairments are among the most frequent symptoms of the disease, some NF1 children did not show cognitive deficits (North, 2000; Ferner et al., 2007).

As a final consideration, note the sample size included in this study is relatively small. This was determined by the very low prevalence of the NF1, thus, the results and implications of this study must be taken with some caution. Additionally, distinct NF1-associated clinical manifestations characterized the sample and no data concerning the tumor suppressor gene inactivation timing or in what cell type's inactivation of the NF1 gene occurs is available. This information would allow selecting more homogenous samples, and would critically influence the understanding of the biological context underlying the cognitive architecture in NF1 and the development of intervention methods for the disease.

## Conclusions

The presence of SLD in the NF1 sample studied is similar to previous reports in this genetic syndrome. Interestingly, different patterns of dissociation between the deficits in basic arithmetical and reading skills were detected. The present study confirmed word decoding deficits and poor number facts retrieval underlying DL and DD, respectively. These cognitive processes were significant predictors of reading and arithmetical attainment. However, deficits in core numeric capacities were not found in the children with DD. This finding suggests that in NF1, deficits in basic number skills are not responsible for arithmetic dysfluency. Rather, this could be explained by general-domain cognitive deficits including defective working memory.

The availability of specific neurocognitive profiles in NF1 would increase the efficiency of screening methods for SLD in genetic populations. Additionally, it would inform the design of individual-based cognitive interventions and pharmacological therapies for these children.

### Author contributions

Conceived and designed the study: Miladys Orraca-Castillo, Nancy Estévez-Pérez, Vivian Reigosa-Crespo. Performed the experiments: Miladys Orraca-Castillo. Analyzed the data: Miladys Orraca-Castillo, Nancy Estévez-Pérez. Wrote the manuscript: Nancy Estévez-Pérez, Miladys Orraca-Castillo, Vivian Reigosa-Crespo.

### Conflict of interest statement

The authors declare that the research was conducted in the absence of any commercial or financial relationships that could be construed as a potential conflict of interest.
